# Human gingival fibroblasts: Isolation, characterization, and evaluation of CD146 expression

**DOI:** 10.1016/j.sjbs.2021.01.053

**Published:** 2021-02-03

**Authors:** Samira Diar-Bakirly, Tarek El-Bialy

**Affiliations:** a Faculty of Medicine and Dentistry - University of Alberta, Mohammed Bin Rashid University of Medicine and Health Sciences, United Arab Emirates; b Faculty of Medicine and Dentistry, University of Alberta, 7-020D Katz Group Centre for Pharmacy and Health Research, Canada

**Keywords:** Gingival fibroblasts, CD146, Mesenchymal stem cells

## Abstract

Gingival fibroblasts (GFs) that exhibit adult stem cell-like characteristics are known as gingival mesenchymal stem cells (GMSCs). Specific mesenchymal stem cell (MSC) markers have not been identified to distinguish GMSCs from GFs. Recently, the cell surface molecule known as cluster of differentiation (CD) 146 has been identified as a potential MSC surface marker. In the present study, we investigated the differentiation potential of GMSCs based on CD146 expression.

GFs were isolated by two techniques: tissue explants or enzymatic digestion. GFs were cultured and expanded then magnetically sorted according to CD146 expression. CD146^low^ and CD146^high^ cells were collected, expanded, and then tested for stem cell markers by flow cytometry as well as osteogenic and chondrogenic differentiation potential. The differentiation of these cells was analyzed after 21 days using histology, immunofluorescence, real-time quantitative PCR (qPCR), and glycosaminoglycan (GAG) to DNA ratio (GAG/DNA) assays. Positive histological staining indicated osteogenic differentiation of all groups regardless of the isolation techniques utilized. However, none of the groups demonstrated chondrogenic differentiation, confirmed by the lack of collagen type II in the extracellular matrix (ECM) of GF aggregates. Our data suggest that identification of gingival stem cells based solely on CD146 is not sufficient to properly carry out translational research using gingival fibroblasts for novel therapeutic methods of treating oral disease.

## 1 Introduction

The gingiva, both anatomically and functionally, is a unique structure with gingival fibroblasts (GFs) as the predominant cells of the gingival connective tissue. The existence of various subpopulations of GFs has been reported (Fournieret al., 2013, Fournieret al., 2010, Phippset al., 1997) However, these subpopulations are phenotypically different, sharing fibroblast-like structures and requiring identical growth conditions *in vitro* (Fournier et al., 2013).

A distinct property of the gingival cells is their role during scarless wound healing. Upon damage to oral tissues, the inflammatory response is manifested by a unique cytokine response from the GFs. At the same time, the GFs display fetal fibroblast-like properties including those related to migration and the production of migrating stimulation factors (Davidet al., 2014, Haekkinen et al., 2000). This healing capacity of the gingiva and its regenerative capacity has resulted in extensive research to identify the resident stem cell population within the gingiva with the ability to self-renew (Politis et al., 2016).

Gingival tissue represents an ideal source of tissue biopsies and GFs due to its accessibility and significantly reduced donor site morbidity compared to other dental tissues (Jinet al., 2015, Mostafaet al., 2008, Zhanget al., 2009). The literature offers overwhelming evidence to support the hypothesis that a subgroup within the GF cell population possesses mesenchymal stem cell (MSC) properties – and are thus called gingival mesenchymal stem cells (GMSCs) (Gardin et al., 2016). Whether sorted (enriched) or unsorted, several studies have demonstrated that these GMSCs are able to differentiate into more than one lineage *in vitro* including osteogenic, chondrogenic, and adipogenic (Fournieret al., 2010, Marynka-Kalmaniet al., 2010, Mitranoet al., 2010, Tanget al., 2011, Zhaoet al., 2015).

In order to identify these cells with stem cell properties, analysis of the ability to self-renew and differentiation potential is required. Several methods towards this goal have been reported including selective growth methods, physical separation, or clonal analysis (Hope & Bhatia, 2011). Centrifugation and cell sorting methods have also been recommended. However, the latter provides more accurate evidence of the cell types involved. The two most widely used methods of cell sorting include fluorescence activated sorting (FACS) and magnetic assisted cell sorting (MACS), providing 75% to more than 95% purity, respectively (Zhu & Murthy, 2013). Both methods employ antibodies that are specific to stem cell markers, which are surface proteins, used to identify these unique populations. Termed as clusters of differentiation, or cluster of designation (CD), protein surface markers can also act as receptors or ligands and are formed during cell development and maturation (Lvet al., 2014, Politiset al., 2016). The majority of the identified stem cell markers are not universal and ongoing research seeks to identify the marker or set of markers that can be used to identify MSCs in different tissues. Notably, expression of CD146 has been reported in almost every type of MSC and has since emerged as the marker of choice for identifying MSCs (Lv et al., 2014).

CD146 is believed to play an integral role in multiple functions related to cell proliferation, development, signal transduction, angiogenesis, cancer metastases, immune response, and cell migration (Harknesset al., 2016, Ouhtitet al., 2009, Wang and Yan, 2013). Additionally, CD146 has been identified as a marker for pericytes, MSCs, endothelial progenitor cells, and osteoblasts and is also expressed during embryonic tissue development (Harknesset al., 2016, Ouhtitet al., 2009, Wang and Yan, 2013). Two reports have demonstrated no effect of CD146 expression on the differentiation potential of MSCs (Espagnolleet al., 2014, Torminet al., 2011). In contrast, other reports show a variety of effects on stem cells, including increased differentiation potential or decreased overall differentiation potential (Harknesset al., 2016, Paduanoet al., 2016). Therefore, the ability of CD146 to be a determinant marker specifically distinguishing GFs from GMSCs remains to be studied. The identification of surface markers and the definitive utility in isolating pure MSC populations are of great value. There is significant demand to characterize additional surface markers and different MSC populations to identify the most reliable MSC marker for use *in vitro* that can also be recommended for future in vivo studies. The current study investigates the role of CD146 to distinguish GMSCs from isolated GFs and assesses the impact of two isolation methods on GF differentiation potential.

## 2 Materials and methods

### 2.1 Isolation and expansion of human gingival fibroblasts

The gingival tissue collection procedure was approved by the University of Alberta Health Research Ethics Board (Pro00001454). Gingival tissues were collected from six healthy adolescent patients’ (3 males, 3 females) interdental papillae during extractions of their first or second premolars as part of their regular orthodontic treatment. All patients read and signed an approved consent form prior to the collection of tissues.

Tissues were weighed (0.18 g–0.21 g) and immediately stored in sterile saline solution for one to four hours before processing. Gingival tissues were washed 10 times in phosphate-buffered saline (PBS) to dilute the oral bacterial flora of the gingival tissue. Following the PBS wash, the tissues were weighed and cut into small pieces of between 1 and 2 mm^2^ using a No. 10 surgical blade (Sigma-Aldrich, Missouri, US). Each sample was divided into two equal portions and one portion was used for each of the two isolation methods (Fournier et al., 2013).

*Enzymatic digestion*. This technique entails incubating the tissue in a collagenase I (2 mg/mL; Worthington Biochemical, Lakewood, NJ, USA) solution for one hour at 37 °C in a 5% CO_2_ incubator. The tissues were then filtered through a 100 μm nylon mesh filter (Falcon, BD Bioscience, NJ, USA) and centrifuged for 10 min at 1500 rpm. The cells were re-suspended in a fresh medium and plated at a density of 10^5^ cell/cm^2^ in sterile 25 cm^2^ tissue culture flasks (Corning, Corning, New York). After 48 h, the medium was replaced. The medium used for culture and expansion of these cells consisted of standard alpha minimum essential medium Eagle (αMEM) supplemented with 10% v/v FBS, 100 mM 4-(2-hydroxyethyl)-1-piperazineethanesulfonic acid (HEPES), 100 U/mL penicillin, 100 μg/mL streptomycin (Sigma-Aldrich, Missouri, US) with the addition of 5 ng/mL of FGF-2 (#PR80001, Neuromics, MN, US). The medium was changed every 2 to 3 days. Once the flask was confluent (after approximately one week of culture), the cells were passaged. At passage 1 (P1), the cells were trypsinized (0.05% w/v Trypsin/EDTA, Invitrogen), counted, and magnetically sorted using CD146 microbead kit (Mitenyi Biotec Cat. no: 130–093-596) following the manufacturer's instructions. At the conclusion of the magnetic sorting procedure, four groups of cells were obtained and then expanded to passage 2 (P2) and passage 3 (P3). The number of cells at P3 was sufficient for experimentation.

*Tissue explants*. The gingival tissue was cut into small pieces and plated over 25 cm^2^ tissue culture flasks and incubated for 48 h, undisturbed, at 37 °C in a humidified incubator with 5% CO_2_. After 48 h, the medium was replaced. Similar to the enzymatic digestion group, the cells at P1 were magnetically sorted and expanded to P2 and P3 until the numbers of cells were sufficient for experimentation.

### 2.2 Phenotypic analysis by flow cytometry

Initially, the expression of CD146 was assessed at the conclusion of the magnetic sorting procedure to ensure proper sorting of CD146^high^ and CD146^low^ cells. Following the culture and expansion of the four experimental groups: Enzymatic-CD146^high^, Enzymatic-CD146^low^, Explant-CD146^high^, and Explant-CD146^low^ stem cell markers CD90, CD105, and CD73, and hematopoietic surface markers CD45 and CD34 along with CD146, were assessed by flow cytometry. 25 × 10^4^ cells were washed with ice-cold FACS buffer (PBS, 0.5% v/v FBS and 0.1% w/v sodium azide) and then incubated for 45 min with primary monoclonal antibodies conjugated to fluorescein isothiocyanate (mAb-FITC) or phycoerythrin (mAb-PE). Fluorescently-conjugated antibodies were CD146-PE, CD90-FITC, CD105-FITC, CD73-FITC, CD34-FITC, and CD45-FITC (BD Biosciences; Catalog numbers: 550315, 555595, 561443, 561254, 555821, 555482). The cells were washed and fixed with 2% v/v paraformaldehyde for 15 min, washed again, and suspended in 1 mL FACS buffer. Isotype-matched controls were incubated with FITC- and PE-conjugated mIgG1. A Fortessa SORP flow cytometer was used to acquire 10^4^ cells. The results of the flow were analyzed using FlowJo software (FlowJo, LLC., Oregon, US).

### 2.3 Osteogenic and chondrogenic differentiation

Osteogenic and chondrogenic media were prepared and used to differentiate the gingival fibroblasts into osteogenic and chondrogenic lineages. For osteogenic induction, 10^5^/mm^2^ cells were cultured in a monolayer on 6-well plates. Three wells were used for staining, while another three were used for gene analysis. Osteogenic medium was composed of Dulbecco′s Modified Eagle′s Medium (DMEM), 100 U/mL penicillin, 100 μg/mL streptomycin, 10% v/v FBS, 0.1 mM ascorbic acid, 10 mM β-glycerophosphate, 10 nM dexamethasone (Sigma-Aldrich, Missouri, US). Ascorbic acid was used to stimulate the synthesis of collagen type I. β-glycerophosphate was added to promote calcium phosphate deposition and dexamethasone was used to stimulate osteogenesis by increasing alkaline phosphatase activity. The medium was changed every 3 to 4 days for a period of 21 days.

For chondrogenic induction, 25 × 10^4^ cells were counted and centrifuged in 1.5 mL tubes. The pelleted cells were incubated at 37 °C with 5% CO_2_ in a chondrogenic differentiation medium composed of DMEM, 365μg/mL ascorbic acid 2-phosphate, 10 nM dexamethasone, 125μg/mL human serum albumin (Sigma-Aldrich, Missouri, US), 10 ng/ml TGFβ3 solution (#cyt-11, ProSpec, New Jersey, USA), 40μg/mL L-proline, and ITS + Universal Culture Supplement Premix (#CACB354352, Corning Discovery Labware II, California, US). The cells within the pellet aggregated to form a spherical shape that did not adhere to the walls of the tube. The medium was changed every 3 to 4 days over a period of 21 days.

### 2.4 Histological analysis

#### 2.4.1 Alizarin red staining

Positive Alizarin red staining indicates the osteogenic differentiation of GFs. The cells from the four experimental groups were cultured in a monolayer as described in the osteogenic differentiation assay. After a period of 21 days, the wells were washed with distilled water twice and fixed for 15 min at room temperature with 10% w/v neutral buffered formalin (Anachemia Canada Inc., Quebec, Canada). Alizarin red staining was used to stain the cultured wells for 10 min. The wells were washed again on a shaker for 15 min. Finally, images were captured and used to qualitatively assess the alizarin-stained mineralized nodules by light microscopy.

#### 2.4.2 Safranin O staining

Safranin O was used following chondrogenic differentiation to detect if any sulphated proteoglycans are produced in the extracellular matrix. After 21 days of chondrogenic differentiation, the pellets were fixed overnight at 4 °C in 10% w/v neutral-buffered formalin. To preserve the cells and increase hydrophobicity, the pellets were dehydrated by immersing them in incrementally higher concentrations of ethanol. The pellets were then embedded in paraffin and cut into 5 μm sections. To detect whether a matrix of sulphated proteoglycans formed within the pellets, the mounted sections of the pellets were stained with 0.01% w/v safranin-O and counterstained with 0.02% w/v fast green (Sigma-Aldrich, Missouri, US). Safranin O staining was used to assess the chondrogenic differentiation of the gingival fibroblasts. Safranin O stains the acidic proteoglycans with an orange-red color. Fast green is a sulphate group which binds strongly to the amino group on protein and stains the non-collagen sites.

#### 2.4.3 Immunofluorescent staining for collagen type I and collagen type II

To identify the absence or presence of the ECM components collagen type I and collagen type II, we performed immunofluorescent analysis. 5 μm sections were deparaffinized after treatment with UltraClear™ solution followed by ethanol and distilled water. Due to the formation of methylene bridges during fixation, the slides were incubated for 30 min at room temperature in an antigen retrieval enzyme, protease XXV (AP-9006–005, Thermo Scientific, Massachusetts, US). To increase the specificity of the antibodies, the slides were incubated in hyaluronidase (H6254, Sigma-Aldrich, Missouri, US) for 30 min at 37 °C. The sections were then incubated in bovine serum albumin (BSA) 5% w/v to reduce non-specific binding of the antibodies. After BSA incubation, the pellet slices were incubated in primary antibodies: rabbit anti-collagen I (CL50111AP-1, Cedarlane, Ontario, Canada) and mouse anti-collagen II (II-II6B3, Developmental Studies Hybridoma Bank, Iowa, US) overnight at 4 °C with a 1:200 dilution. The preceding step was followed by incubation with a fluorescently-conjugated secondary antibody at a dilution of 1:200 with goat anti-rabbit IgG (H&L Alexa Fluor 594, Abcam, UK) for collagen type I and goat anti-mouse IgG (H&L Alexa Fluor 488, Abcam, UK) for collagen type II. The sections were stained with DAPI (4′, 6-diamidino-2-phenylindole, Cedarlane) to stain the cell nuclei, and mounted with a 1:1 glycerol-PBS solution. Immunofluorescent images were visualized by an Eclipse Ti-S microscope (Nikon Canada, Ontario, Canada).

### 2.5 Biochemical analysis for gingival fibroblast chondrogenesis

After 21 days of chondrogenic induction using the chondrogenic medium, the cells in the pellet were analyzed to detect specific glycosaminoglycan (GAG) content, indicative of production of biomolecules contained in the cartilaginous extracellular matrix after chondrogenic differentiation. After washing the pellets with PBS, 250 μl Protease K (1 mg/mL in 50 mM Tris with 1 mM EDTA, 1 mM iodoacetamide, and 10 mg/mL pepstatin A; Sigma-Aldrich, Missouri, US) was used to digest the pellet at 56 °C overnight. The GAG content was measured spectrophotometrically after using 1,9-dimethylmethylene blue and chondroitin sulphate (Sigma-Aldrich, Missouri, US) as a standard. The DNA content was determined using the CyQuant cell proliferation assay kit (Invitrogen, Ontario, Canada) using the supplied bacteriophage λ DNA as a standard.

### 2.6 RNA extraction and real-time polymerase chain reaction (RT-PCR) analysis

Genes that are related to osteogenic differentiation and dentinogenesis were compared across the four experimental groups and included: 1) runt-related transcription factor 2 (*RUNX2*), essential for osteoblast differentiation; 2) alkaline phosphatase liver/bone/kidney type (*ALPL*), a metalloenzyme expressed during osteogenesis (Pilipchuk, 2018); 3) osteocalcin (*OCN*), which constitutes the majority of noncollagenous bone matrix proteins, is considered a late osteogenic marker, and has recently been found to play a role in transcription factor regulation during mineralization (Tsao et al., 2017); 4) collagen type IA1 (*COLIA1*), the most abundant organic component of bone ECM and is believed to play an important role in enhancing osteogenesis through MSC integrin –collagen type I binding (Lozito et al., 2009) dentin sialophosphoprotein (DSPP), abundant in odontoblast cells and plays an important role in mineralization. DSPP was evaluated as a marker for odontogenic differentiation (Giacaman et al., 2016)Trizol (Life Technologies) was used to extract the RNA from the monolayer osteogenic cultures. The RNA concentrations were measured using a NanoDrop-2000C Spectrophotometer (Thermo Fisher Scientific, Delaware, US). cDNA was then synthesized following the reverse transcription reaction by using 1 μg of Oligo dT (Promega, Wisconsin, US). Dilutions of 1:10 were prepared from the samples to be used in real-time PCR (qPCR). A 10 μl real-time reaction mixture was prepared by adding 3 μl of cDNA, 1 μl each of forward and reverse primers and 5 μl of Takyon™ No Rox Probe MasterMix dTTP Blue (Eurogentec North America, Inc., California, US). Primer sequences are shown in Table 1. The dilutions were then suspended in the 96-well block of the CFX real-time PCR detection system. Hypoxanthine phosphoribosyltransferase 1(HPRT1), Ribosomal Protein L13a (RPL13), and Tyrosine 3-Monooxygenase/Tryptophan 5- Monooxygenase Activation Protein Zeta (YWHAZ) were used as internal controls in each run. These three latter reference genes were used for the accurate quantification of data. Fluorescence data were obtained and plotted against cycle number and then analyzed using CFX connect software.

**Table 1:** Primers sequences for genes of interest.

**Gene**	**Forward**	**Reverse**
**Osteocalcin (*OCN*)**	AATCCGGACTGTGACGAGTTG	CCTAGACCGGGCCGTAGAAG
**Alkaline Phosphatase (A*LPL*)**	CCTGGCAGGGCTCACACT	AAACAGGAGAGTCGCTTCAGAGA
**Runt related transcription factor 2 (*RUNX2/CBFA1*)**	GGAGTGGACGAGGCAAGAGTTT	AGCTTCTGTCTGTGCCTTCTGG
**Collagen type I alpha 1 chain**	GCCTCGGAGGAAACTTTGC	TCCGGTTGATTTCTCATCATAGC
**Dentin sialophosphoprotein (*DSPP*)**	TGGGCAAAGGCAATGTCAA	TGGCCAGGTCCTTCTATGTTG

To determine the relative expression of the genes of interest, we used three reference genes as normalizers. After determining CT values, the difference between the reference and target gene CT values is calculated. The relative expression of the target gene is then determined by using the 2^-Δ CT^ formula to compare and evaluate the osteogenic gene expression between groups.

### 2.7 Statistical analysis

The data presented in the graphs represent the average and standard deviation in each group for the six donors. Statistical analysis was performed using IBM® SPSS® Version 23.0 (IBM Corp., Armonk, N.Y., USA) and Excel 2016 (Microsoft, Washington, US). A normality test to assess the distribution of the data was performed using the Shapiro-Wilk test. A repeated two-way ANOVA was used to determine whether there was any interaction between the isolation method and sorting groups followed by further assessment of the primary effect of the isolation method and CD146 expression. Statistical significance was considered when p < 0.05.

## 3 Results

### 3.1 Magnetic separation using CD146 magnetic beads

**Fig. 1:**
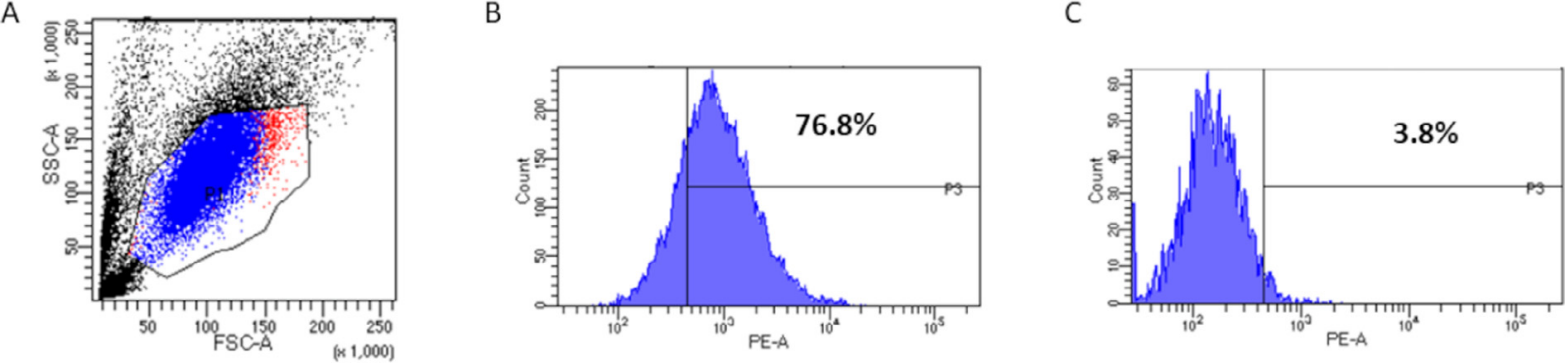
**CD146 expression immediately following magnetic sorting.** (A) Forward and side scatter of the GF population. (B) CD146 was expressed in 76.8% of the GF population of CD146^high^ cells. (C) CD146 was expressed in 3.8% of the GF population of CD146^low^ cells.

Fig. 1A demonstrates GFs population with high expression in the CD146^high^-sorted group (Fig. 1B) and low expression in the CD146^low^-sorted group (Fig. 1C).

### 3.2 Phenotypic analysis by flow cytometry

**Fig. 2:**
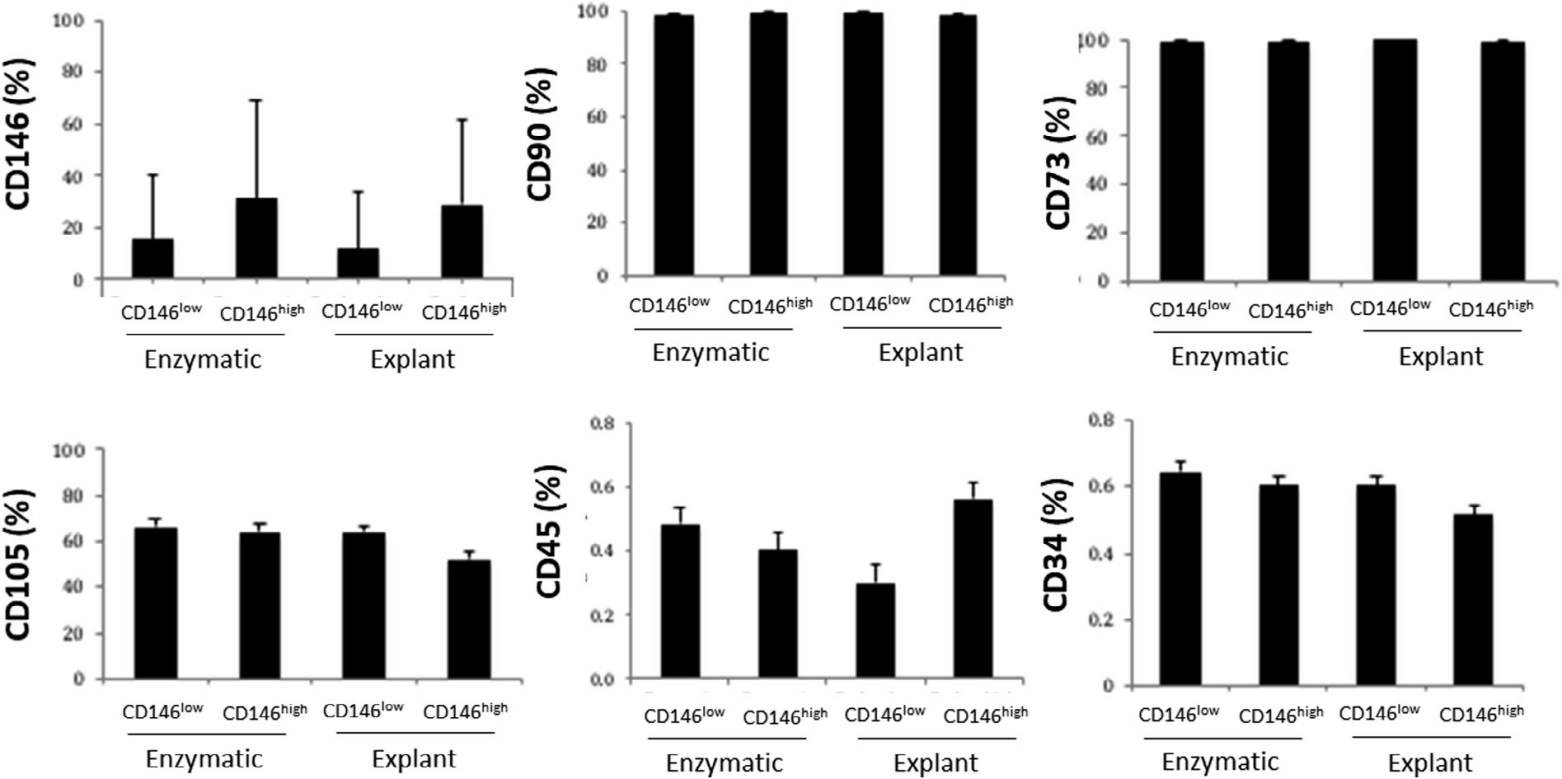
**Flow cytometry analysis of CD surface markers.** 25 × 10^4^ cells were washed with ice-cold FACS buffer and then incubated for 45 min with primary monoclonal antibodies. The bars represent the average expression of surface markers: CD146, CD90, CD70, CD105, CD34, and CD45 in all four experimental groups. Values are expressed as the average ± SD. The expression of CD 146 in both CD146^low^ and CD146^high^ groups shows inconsistent and great variation between individuals.

All patients exhibited low expression of CD146 in the CD146^low^ groups compared to the CD146^high^ groups whether the enzymatic or explant method was used. All four experimental groups expressed the positive MSC markers CD90, CD105, and CD73 but not the hematopoietic markers CD45 or CD34. No statistical significance was detected for any marker (Fig. 2).

### 3.3 Osteogenic assays

#### 3.3.1 Alizarin red staining

**Fig. 3:**
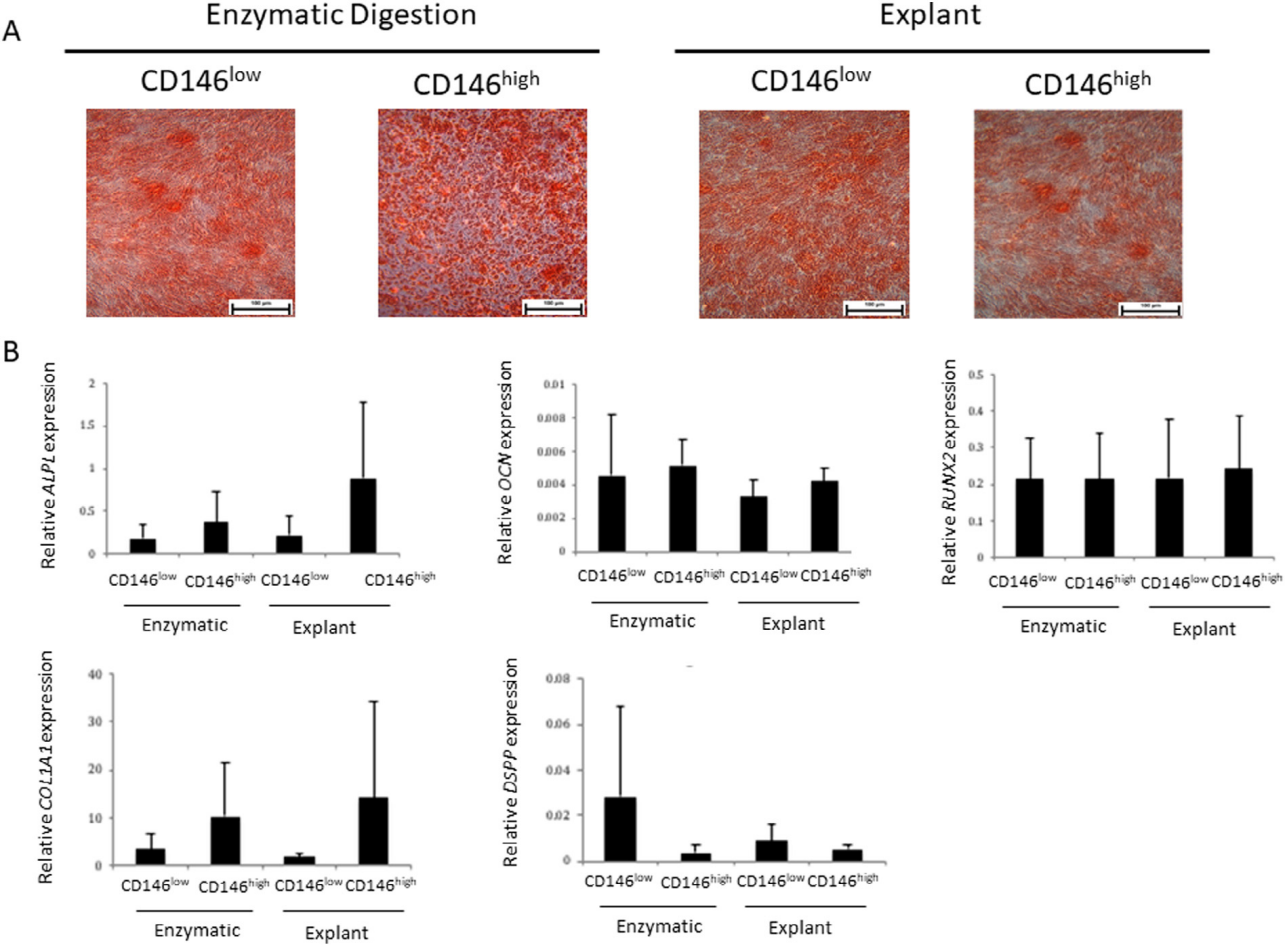
**Osteogenic differentiation of GMSCs.** A) Mineralization nodules were detected with alizarin red after 21 days of osteogenic differentiation. Scale bar (black) represents 100 µm. B) Relative osteogenic gene expression for [alkaline phosphatase (ALPL), osteocalcin (OCN), runt related transcription factor 2 (RUNX2), type I collagen (COL1A1), and dentine sialophosphoprotein (DSPP)] (n = 6/group).

As evidence of the osteogenic differentiation potential of the gingival fibroblasts, all patient samples exhibited alizarin red staining in all isolation type groups (Fig. 3A).

#### 3.3.2 Gene analysis following osteogenic differentiation

The expression of relative osteogenic genes was compared to further investigate any differences between the capacities of the four experimental groups towards osteogenic differentiation. No significant differences were observed in the expression of *RUNX2, OCN*, *OPN*, *COLIA1,* or *DSSP* among the four experimental groups (Fig. 3B).

### 3.4 Chondrogenic assays

#### 3.4.1 Safranin O staining

**Fig. 4:**
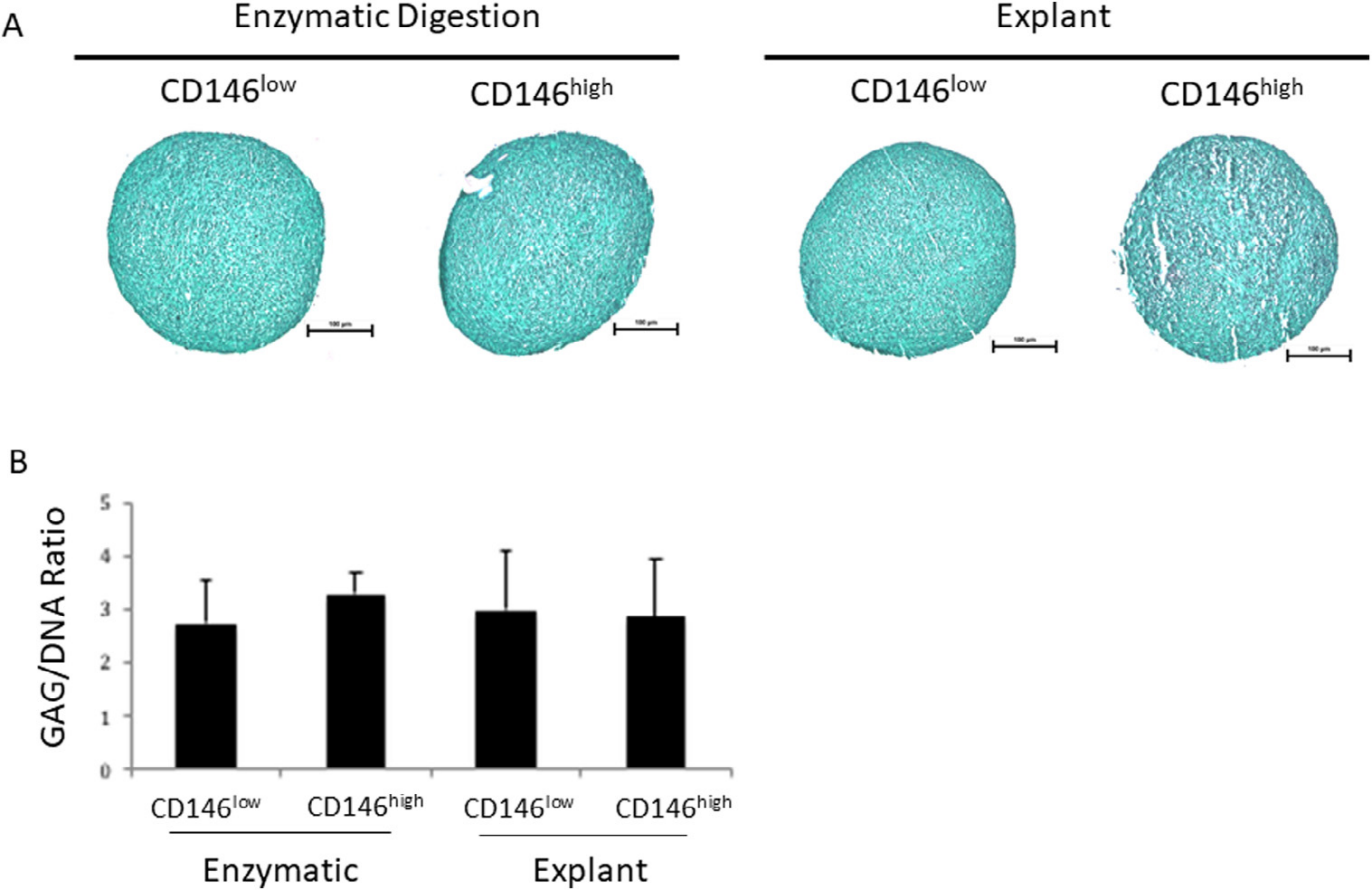
**Chondrogenic differentiation of GMSCs.** A) No evidence of safranin O stain (pink) that for sulphated proteoglycans. Scale bar (black) represents 100 µm. B) Pelleted cells from all patients (N = 6) in all experimental groups were analysed by RT-PCR to generate GAG, DNA, and GAG/DNA ratios. Values expressed as mean ± SD.

All patient samples did not exhibit positive Safranin O stain, with no significant difference among the four experimental groups (Fig. 4A), suggesting that these cells were not being driven towards chondrogenesis.

#### 3.4.2 Biochemical analysis of gingival fibroblast chondrogenesis

No significant difference was observed in the ratio of GAG/DNA among the four experimental groups (Fig. 4B).

### 3.5 Immunofluorescent staining for collagen type I and collagen type II

**Fig. 5:**
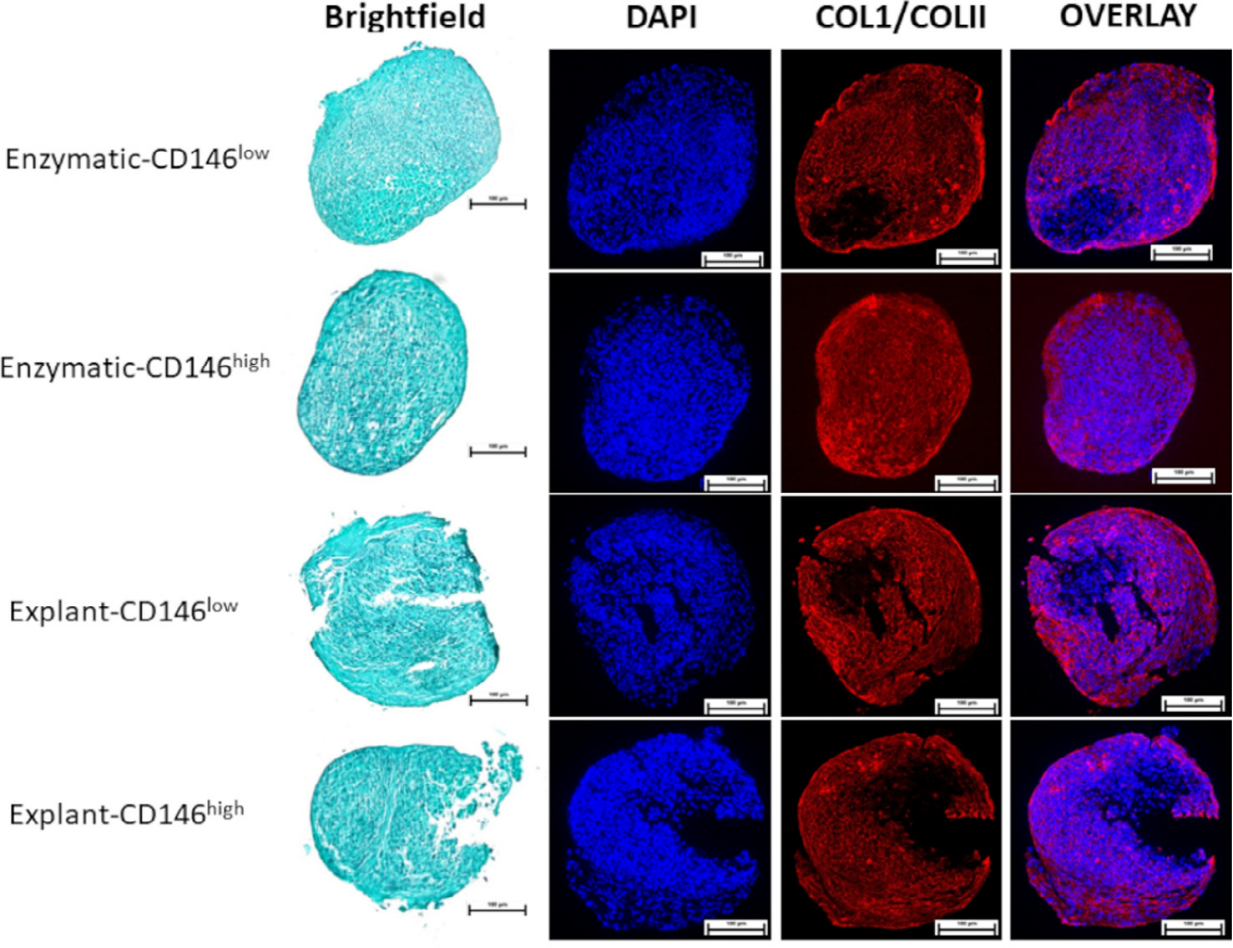
**Immunofluorescent detection of chondrogenesis.** Representative images from one patient sample fluorescently labelled with antibodies for collagen type I (red) and type II (green), and DAPI (blue). Scale bar represents 100 µm.

DAPI staining indicated uniform distribution of cells while collagen type I was produced throughout the entirety of each pellet with no outstanding variations observed among the four groups (Fig. 5). Collagen type II was not detected in the extracellular matrix of the pellets, a result that aligns with the low GAG content, negating the presumed chondrogenic differentiation potential in the gingival fibroblasts.

## 4 Discussion

The current study utilized gingival tissue to isolate and expand GFs, specifically due to its ease of access and the promising multilineage potential of these cells (Fawzy El-Sayed & Dörfer, 2016, Fournieret al., 2013, Hyneset al., 2012, Mostafaet al., 2011, Zhaoet al., 2015). More importantly, we attempted to identify GMSCs within GF cultures. Since MSCs are considered to be heterogeneous cell populations within the same isolated culture, the identification of specific GMSC marker(s) is essential for tissue engineering treatments, not only for dental tissue regeneration, but potentially other organ systems (Harkness et al., 2016).

Starting with the idea that CD146 is a putative MSC marker (El-Sayedet al., 2015, Espagnolleet al., 2014, Harknesset al., 2016, Paduanoet al., 2016, Torminet al., 2011, Zhuet al., 2013) and its expression in MSCs might increase the differentiation capacity and thus the stem cell properties (Bakshet al., 2007, Russellet al., 2010), we wanted to determine if the CD146 expression in GFs correlates to higher stemness. First, we enriched the GFs population by using the MACS. Then, we attempted to differentiate both CD146^high^ and CD146^low^ cultures into two lineages: osteogenic and chondrogenic.

In our study, CD146^low^ and CD146^high^ cultures express similar levels of MSC-positive marker expression, with the expected negative expression of hematopoietic markers (CD34 and CD45), regardless of the isolation method. Moreover, the loss of CD146 expression in three of our six samples of the CD146^high^ expression groups was similar to previously published work, which showed a loss of CD146 expression from P1 to P6 (Paduano et al., 2016). The results of this study are in contrary to previous research that showed that CD146 maintained high expression after passage 6 (Harkness et al., 2016). This could be attributed to the different tissue sources used (i.e., bone marrow versus gingival interdental tissue). It has also been reported that CD146 expression was inconsistent in the cells that were studied and greatly affected by the anatomical site from which the tissues were derived (Espagnolleet al., 2014, Torminet al., 2011). Taken together, there is conflicting evidence in the published literature which currently prevents conclusions regarding the role of CD146 in MSC characterization.

Our results revealed that the osteogenic differentiation potential of both CD146^high^ and CD146^low^ cultures was similar when assessed phenotypically following alizarin red staining. Quantitative analysis bore similar results: neither CD146 expression nor the isolation methods had any effect on *ALPL, OCN, COLIA1, or DSPP* gene expression. Studies using dental-derived MSCs have reported contradictory results with regard to the differentiation potential between CD146^low^ and CD146^high^ cultures. Several studies have demonstrated increased differentiation potential of MSCs for CD146^high^ cultures as opposed to CD146^low^ cultures (Tavangaret al., 2017, Xuet al., 2008, Zhuet al., 2013). In addition, (Sorrentino et al., 2008) reported an increased differentiation potential and stem cell marker expression among CD146^high^ cells. However, this study used bone marrow-derived stem cells (BMSCs) and made no comparison to CD146^low^ cells (Sorrentino et al., 2008).

Two additional studies using BMSCs reported a similar differentiation potential for both CD146^low^ and CD146^high^ cultures. Both studies obtained their cells from fresh bone marrow aspirate and healthy donors and both used basic MSC expansion media without the addition of growth factors (Espagnolleet al., 2014, Torminet al., 2011). Furthermore, a recent study reported no difference in osteogenic gene expression between CD146^high^ and CD146^low^ cultures (Harkness et al., 2016). It is important to note that publications attempting to replicate their data were unable to corroborate these findings. Both high- and low-expressing groups were able to lay bone but the CD146^low^ cultures differentiated and laid significantly more bone compared to the CD146^high^ cultures, which formed more bone marrow. The authors linked their finding to the heterogeneity of the MSC populations - either mature MSCs (laid more bone) or immature MSCs (laid more marrow) (Harkness et al., 2016). In another study, GMSCs were sorted by El-Sayed *et al*. according to STRO-1 and CD146 expression and concluded that CD146^low^ cells possessed superior osteogenic differentiation potential (El-Sayed et al., 2015). Additional evidence supports superior osteogenic potential in CD146^low^ cultures despite a difference in CD146 expression after passaging (Paduano et al., 2016). The role of CD146 has also been explored in other dental stem cells, including human dental pulp stem cells (DPSCs), stems cells from human exfoliated deciduous teeth (SHEDs), and periodontal ligament stem cells (PDLSCs) (Chalisserry et al. 2017). Despite ubiquitous expression of CD146 across these cell types, findings regarding the functional impact on stemness, differentiation potential, and proliferation capacity have been contradictory. In summary, it remains unclear whether the expression level of CD146 has any substantial effect on the MSC phenotype.

Our results indicate a high osteogenic differentiation potential in all four experimental groups of isolated cells, unaffected by the isolation or enrichment method. However, the quantitative assessment of GAG production was not statistically different among all groups and did not support chondrogenic differentiation. In addition, our pellets have shown similar qualitative results following safranin O staining and none of the pellets displayed evidence of sulphated glycosaminoglycans. To further characterize the ECM collagenous component produced in the cellular aggregates, immunofluorescence results show high and equal production of collagen type I in all pellets and no collagen type II production was detected. Taken together, our results demonstrate that the sorted GFs did not undergo chondrogenic differentiation and suggest that the isolated and sorted GFs that express CD146 protein may be osteoprogenitor cells rather than mesenchymal stem cells that possess multilineage differentiation potential. It is of importance to note that sorting GFs based on a combination of proteins markers expressed rather than the solemn expression of CD146 might have led to selection of a population of cells that might identify as MSC and thus shows different stemness and differentiation potential. Further research in this field is still necessary to identify the markers that can distinguish the GMSC population residing within the GFs.

As previously mentioned, extractions of dental MSCs from different anatomical locations have been performed using either tissue explants or enzymatic digestion. Here, we tested whether the isolation method had any effect on the expression of MSC markers or CD146 and compared the osteogenic/chondrogenic differentiation potential. We report that, regardless of the isolation and culture method, the expression of MSC markers, including CD146, and the differentiation potential remained unchanged in our isolated populations. To our knowledge, this is the first study that has considered the effects of isolation methods on MSC marker expression and differentiation potential of GFs derived from gingival tissue. The importance of this experiment demonstrates that a consistent protocol will be required to advance MSCs for therapeutic or regenerative purposes.

Upon establishment of a validated protocol for their isolation, GMSCs may serve as an alternative to bone marrow and/or dental pulp stem cells in dental and dentofacial regeneration and tissue engineering. GMSCs have been shown to repair skin wounds and to treat patients with rheumatoid arthritis and other immune diseases (Chen et al., 2013), effects attributed to the angiogenic, anti-inflammatory, and immunomodulatory properties of the cell type. GMSCs modulate the recruitment of mast cells and neutrophils to sites of tissue injury (Cha et al., 2017) and induce polarization of macrophages from the inflammatory M1 to the reparative M2 phenotype (Fournier et al., 2013). The restorative capacity of GMSCs has been demonstrated in preclinical species and in patients. When loaded onto either an inorganic porous matrix of deproteinized bovine bone or an organic collagen matrix, GMSCs corrected periodontal defects in a porcine model (Fawzy El-Sayedet al., 2012). Similarly, biopsy-derived GMSCs increased gingival width in patients when used over a non-woven matrix (Prato et al., 2003). Further advancements in scaffold materials may expedite the implementation of GMSCs in the clinical setting.

A current limitation of GMSCs precluding therapeutic application is their heterogeneity with regard to cell surface antigen expression, morphology, proliferation rate, and response to inflammatory cytokines. Specific subtypes of GMSCs can be identified in GF-derived cultures. The fibroblasts multiply and form distinct colonies from single cells or multi-cell aggregates, colonies which subsequently display varying self-renewal capacities following expansion (Fournier et al., 2013). These divergent phenotypes, again, undermine the need for optimal isolation, differentiation, characterization, and handling protocols such that the regenerative and reparative qualities of distinct lineages can be leveraged in future clinical trials.

We isolated GMSCs from GF populations using the two most common methods, the explant and enzymatic digestion methods. We also enriched the cell populations for CD146 expression. Neither isolation nor CD146 expression demonstrated any significant effect on the expression of MSC markers or the differentiation potential of these cells. Thus, our data suggest both isolation methods are sufficient to isolate GFs and that CD146 expression does not appear to be a specific MSC surface marker to isolate or enrich the MSC population from the total GF pool. These results warrant pursuit of novel cell surface markers –independently or in combination with CD146 – that are more predictive of stemness and differentiation capacity to enable the use of GFs and other dental stem cells in the therapeutic setting.

Our study was not without limitations. It is possible that statistical significance was not achieved due to the relatively small sample size. Furthermore, the inclusion of experimental control groups that did not receive FGF-2 treatment was lacking. Moreover, the cells were passed with magnetic beads only once in the extraction tube. Passing the cells twice in the tube or using flow assisted cell-sorting machines, which yield more accurate and purer populations of cells, may have presented superior methods for sorting cells. It is also possible that the isolation methods could be optimized, which would be the subject of future studies.

Based on our results and limitations, the following recommendations can be explored in future studies: Incessant attempts to test different combinations of MSC markers and hematopoietic antigens in the GFs population to identify and isolate the optimal GMSC population should continue. Lack of expression of markers such as HLA-DR, with the expression of other proposed MSC markers as STRO-1 and CD271, could allow optimal use of GMSCs in future translational, clinical, and therapeutic applications (Ahmedet al., 2020, Iozonet al., 2020, Widowatiet al., 2019). Moreover, more studies could be conducted to understand potential difference of differentiation and surface marker expression based on gender, age, race, and health status of the individual. Furthermore, evaluate the potential of cryopreservation of the isolated GMSCs while maintaining their therapeutic potential. Once the GMSC population has been identified and defined, further in vivo study models would help to establish potential therapeutic guidelines.

## Declaration of Competing Interest

The authors declare that they have no known competing financial interests or personal relationships that could have appeared to influence the work reported in this paper.
